# Post-myocardial infarction left ventricular septal dissecting aneurysm: a case report

**DOI:** 10.1186/s40792-021-01141-7

**Published:** 2021-02-27

**Authors:** Yuji Kamikawa, Takeki Ohashi, Masao Tadakoshi, Akinori Kojima, Hirotaka Yamauchi, Kaoru Hioki, Takanori Hishikawa, Souichirou Kageyama

**Affiliations:** 1 Cardiovascular Surgery Department, Nagoya Tokushukai General Hospital, 2-52 Kozoji-cho kita, Kasugai, Aichi 487-0016 Japan; 2 Cardiovascular Surgery Department, Sendai Tokushukai Hospital, 15 Kagosawa, Izumi-ku, Sendai, Miyagi 981-3131 Japan

**Keywords:** Left ventricular septal aneurysm, Post-myocardial infarction, Ventricular septal dissection

## Abstract

**Background:**

Post-infarction perforation of the ventricular septum is recognized as a major complication of post-myocardial infarction. However, post-infarction ventricle dissection is seldom reported, as the ventricular shunt often accompanying this condition is a significant cause of cardiogenic shock. We encountered a rare case of ventricular dissection unaccompanied by a shunt, which caused a state of shock.

**Case presentation:**

A 67-year-old man was diagnosed with acute myocardial infarction with a left ventricular oozing rupture. The occlusion of the left anterior descending artery was aspirated, followed by drainage of the pericardial bleeding and hemostasis of the left ventricle. After 15 h, he presented with sudden cardiogenic shock requiring extra-corporeal membrane oxygenation. The transesophageal echocardiogram showed a left ventricular septal aneurysm. Five days later, he underwent an operation, in which a ventricular septal wall dissection with a tear-forming large pseudoaneurysm was found. The tear was closed with a patch. He was weaned off extra-corporeal membrane oxygenation the next day. Αfter 4 months, he was discharged in a stable condition.

**Conclusions:**

Recognizing and identifying the cause of cardiogenic shock after myocardial infarction is crucial to provide the best treatment and surgical approach. Ventricular septal dissection should be considered, in addition to the usual complications, such as possible papillary muscle rupture, cardiac rupture, and perforation of the interventricular septum.

## Background

Post-myocardial infarction ventricular rupture is a major serious complication following myocardial infarction. The rupture usually penetrates the interventricular septum, forming a shunt, which leads to heart failure. In rare cases, a shunt is created by peeling and perforation of the interventricular septum, termed ventricular dissection [1–4]. However, there have been few reports of post-myocardial infarction ventricular septal dissection unaccompanied by a shunt [5–7]. We herein report the case of a patient with post-myocardial infarction ventricular septal dissection forming a septal aneurysm that caused cardiogenic shock. The defect was surgically repaired and the patient recovered.

## Case presentation

**Fig. 1:**
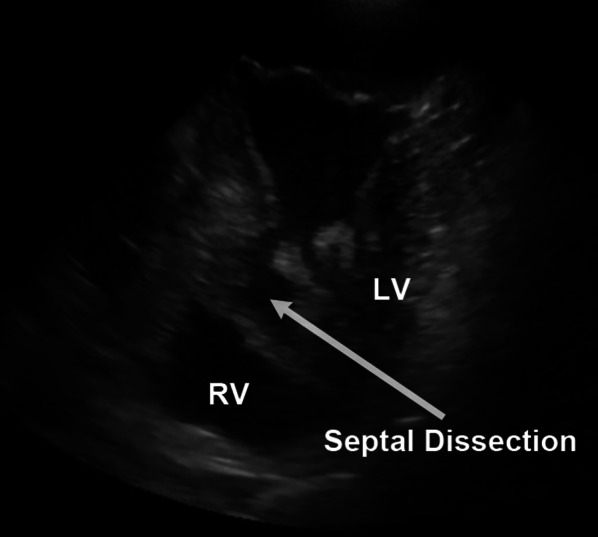
Transesophageal echocardiogram. A ventricular septal dissection is shown with communication to the left ventricle via a small orifice-generating aneurysm. *LV* left ventricle, *RV* right ventricle

**Fig. 2:**
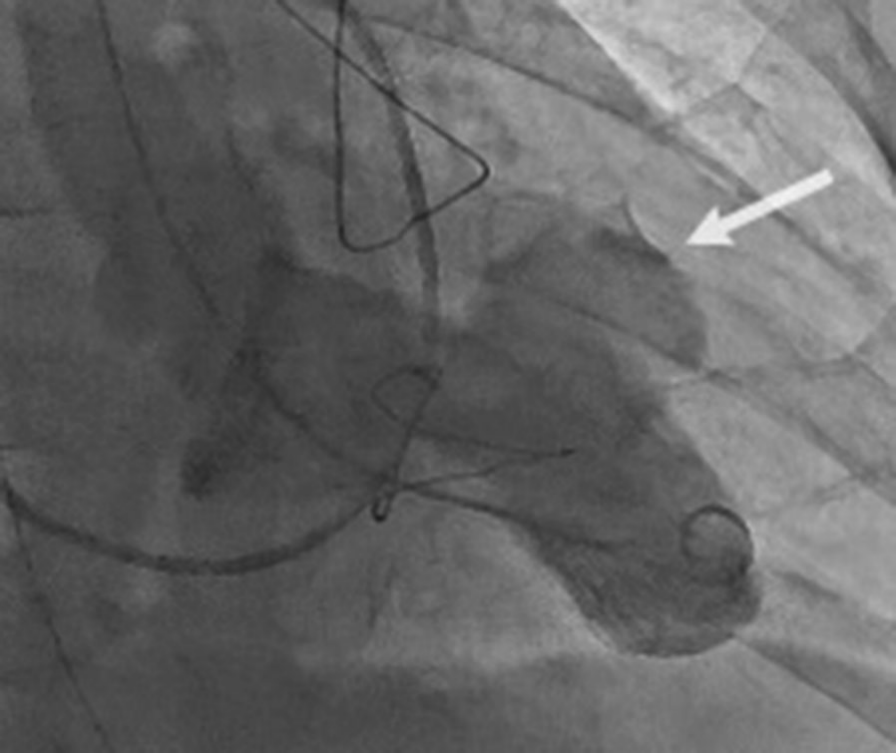
Left ventriculogram revealing a saccular aneurysm-like protrusion at the ventricular septum (white arrow)

**Fig. 3:**
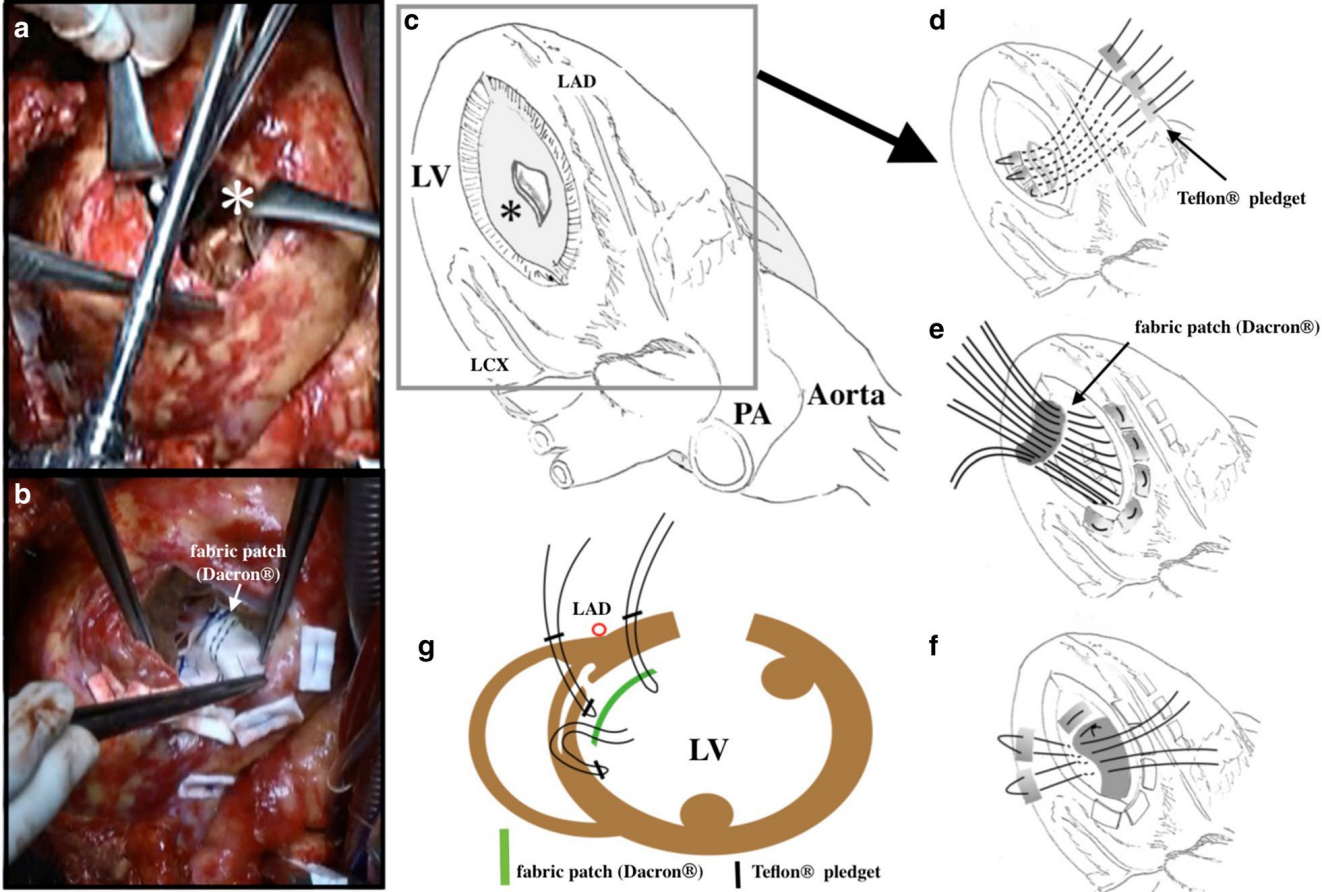
**a** Left ventriculotomy showing a small orifice at the ventricular septum. From this orifice, a septal dissecting aneurysm was formed. **b** Obliteration of the septal dissection. The left ventricle patch was placed over the infarcted septal wall including tear and sutured with pledget 3-0 polypropylene. **c** The orifice of the septal dissection was located at the middle part of the ventricular septum. **d** A large suture was passed through the right ventricle from the septum using 3-0 polypropylene with Teflon felt. **e**, **f** A fabric oval patch was seated down on the ventricular septum and sutured with pledget 3-0 polypropylene. **g** A schematic picture of the surgical procedure. The green bar indicates the fabric patch. The small black bars indicate the Teflon pledgets. ( ∗) indicates the orifice of the septal dissection. *LV* left ventricle, *LAD* left ascending coronary artery, *LCX* left circumflex coronary artery, *PA* pulmonary artery

A 67-year-old man presenting with chest discomfort and nausea was transferred to the nearby hospital. He was diagnosed with acute myocardial infarction; an intra-aortic balloon pump (IABP) was inserted, and he was taken to the catheter room. His angiogram findings revealed left anterior descending coronary artery occlusion with a thrombus that was aspirated immediately. Echocardiography revealed a pericardial effusion suggestive of left ventricular rupture, and he was referred to our unit for surgery. The patient had been consulting a local physician for hypertension, diabetes mellitus, and hyperlipidemia. He had a 47-year history of daily smoking. His blood pressure was 143/92 mmHg, and the pulse rate was 86 beats/min. His blood oxygen saturation (SpO_2_) level was 98% with 8 L/min of oxygen therapy. His chest radiography findings showed remarkable cardiomegaly with pulmonary congestion. The 12-lead electrocardiogram findings demonstrated sinus rhythm with ST elevation and poor R progression in leads V1–4. Laboratory findings showed that the cardiac enzymes, creatine kinase and creatine kinase-MB, were elevated at 196 and 17 IU/L (peak level of creatine kinase-MB), respectively, and reached 2223 and 14 IU/L 3 days later. Troponin I level was remarkably elevated at 19.26 ng/mL on admission. A median sternotomy was performed, and he underwent drainage of the pericardial bleeding and hemostasis of the left ventricular oozing rupture without cardiopulmonary bypass. A tissue-sealing seat was applied to the left ventricle epicardium using human fibrinogen, thrombin, and aprotinin. During surgery, we performed a transesophageal echocardiogram (TEE), which revealed a small slit formation of the ventricular septum; however, it showed no shunt between the right and left ventricles. The patient was transferred to the intensive care unit in a stable condition. A transthoracic echocardiogram showed akinetic regions of the large anterior segment and apex of the left ventricle. Although the slitted interventricle septum was depicted with fluttering motions, no shunt was indicated (Additional file 1: Video S1). However, 15 h later, he presented with sudden cardiogenic shock requiring extra-corporeal membrane oxygenation (ECMO). The patient could not be weaned from ECMO, and a TEE performed 5 days later showed the new onset of a left ventricular septal aneurysm (Fig. 1, Additional file 2: Video S2). The left ventriculogram also revealed a saccular aneurysm-like protrusion at the ventricular septum (Fig. 2), which caused severe left ventricular acute failure and ECMO support dependence. Then, the patient was taken to the operating room for surgical repair. Re-sternotomy was performed, and cardiopulmonary bypass was instituted from the superior and inferior veins to the ascending aorta. A left ventricular vent tube was inserted via the right upper pulmonary vein. The ascending aorta was clamped and the heart was arrested with antegrade cardioplegia. Then, the left ventricle was incised at the lateral portion of the left anterior descending coronary artery. The ventricular septal wall had a dissection with a single tear into the left ventricle, forming a large aneurysm (Fig. 3a, c). The septal dissection was obliterated with a large suture through the right ventricle from the septum using 3-0 polypropylene suture with Teflon felt (Fig. 3d). Inside the left ventricle, a 4 × 6-cm oval fabric patch was placed over the infarcted septal wall, including the rupture, and was sutured with pledget 3-0 polypropylene (Fig. 3b, e–g). An additional aortocoronary bypass graft with a saphenous vein graft was performed. Cardiopulmonary bypass was weaned off with ECMO and IABP. Hemostasis was secured, and the surgery was completed. The patient was weaned off ECMO on postoperative day (POD) 1. The IABP was removed on POD 5, and the patient was extubated on POD 15. He was complicated by a small left temporal lobe infarction. After 4 months, he was transferred to another hospital for rehabilitation and was discharged 1 year later.

## Conclusions

There have been many reports regarding post-myocardial infarction complications, such as left ventricular rupture, ventricular septal perforation, and papillary muscle rupture [1]. Some reports have described cases of post-myocardial infarction ventricular septal dissection associated with septal perforation [2–4]. However, there have been few reports of post-myocardial infarction ventricular septal dissection forming a septal aneurysm without a shunt [5, 6]. Furthermore, to the best of our knowledge, only one case report described a case of a patient who underwent surgical repair of ventricular dissection unaccompanied by a shunt [7]. This type of pathology is different from other types of post-myocardial infarction complications, such as left ventricular aneurysm or pseudoaneurysms, which we encountered frequently. In this case, aneurysmal formation involved a dissected septal wall (referred to as the pseudo-lumen) communicating to the left ventricle. After the surgery for left ventricular oozing rupture, the patient was in a good and stable condition. However, an intraoperative TEE showed a left ventricular septal slit. After 15 h, he suddenly presented with cardiogenic shock requiring veno-arterial ECMO, which suggested that the septal infarcted wall slit had expanded into a dissection with a large pseudo-lumen aneurysm. Then, ventricular septal perforation was suspected; however, frequent echocardiograms showed no left to right ventricular shunt. Instead of developing into a left ventricular septal perforation, the ventricular septal dissection expanded and communicated to the left ventricle via a small orifice. We assumed that the septum was doubly dominated by the intact right coronary artery and impaired the left anterior descending coronary artery; thus, it created shearing forces at the septum border to cause septal dissection. Thus, the dissected ventricular septal wall became an aneurysm, causing acute heart failure. A left ventriculogram was performed to determine the diagnosis, and a large protruding pseudo-lumen aneurysm was found. We excluded the large pseudo-lumen aneurysm, and the patient’s cardiac condition recovered surprisingly.

To date, there are few anatomical and clinical reports of septal dissection without shunting from the interventricular septum after myocardial infarction. Furthermore, it is extremely rare for a case of ventricular dissection without a shunt to be shocked and successfully repaired surgically. Although the method of surgical repair is simple, anatomical and hemodynamic changes in the remaining interventricular septal pseudo-lumen aneurysm and related complications remain unknown and require close follow-up examination.

## Supplementary Information

**Additional file 1.** **Video S1.** A transthoracic echocardiogram after first surgery showing akinetic regions of the large anterior segment of the left ventricle and a color Doppler image showing the slitted interventricle septum with fluttering motions and no shunt.

**Additional file 2.** **Video S2.** Intraoperative transesophageal echocardiogram showing a left ventricular septal aneurysm causing acute heart failure.

## Data Availability

All data underlying the results are available as part of the article and no additional source data are required.

## Abbreviations

ECMO: Extra-corporeal membrane oxygenation
IABP: Intra-aortic balloon pump
POD: Postoperative day
TEE: Transesophageal echocardiogram
